# Generation of a Focused THz Vortex Beam from a Spintronic THz Emitter with a Helical Fresnel Zone Plate

**DOI:** 10.3390/nano13142037

**Published:** 2023-07-10

**Authors:** Xiaoqiang Zhang, Yong Xu, Bin Hong, Fan Zhang, Anting Wang, Weisheng Zhao

**Affiliations:** 1 Hefei Innovation Research Institute, School of Integrated Circuit Science and Engineering, Beihang University, Hefei 230013, China; xqzhang@buaa.edu.cn (X.Z.); fanzhang@buaa.edu.cn (F.Z.); weisheng.zhao@buaa.edu.cn (W.Z.); 2 Anhui High Reliability Chips Engineering Laboratory, Hefei 230013, China; 3 Department of Optics and Optical Engineering, University of Science and Technology of China, Hefei 230026, China; atwang@ustc.edu.cn

**Keywords:** THz vortex beam, helical Fresnel zone plate, spintronic THz emitter, tight focusing

## Abstract

Similar to optical vortex beams, terahertz (THz) vortex beams (TVBs) also carry orbital angular momentum (OAM). However, little research has been reported on the generation of TVBs. In this paper, based on the detour phase technique, we design a series of spintronic terahertz emitters with a helical Fresnel zone plate (STE-HFZP) to directly generate focused TVBs with topological charges (TCs) of *l* = ±1, ±2 and ±3, respectively. The STE-HFZP is a hybrid THz device composed of a terahertz emitter and a THz lens, and it has a high numerical aperture (NA), achieving subwavelength focal spots. Its focus properties are surveyed systemically through accurate simulations. This STE-HFZP can also generate focused TVBs with higher order TCs. More importantly, the components of the focused electric field with OAM make up the majority of the intensity and have potential applications in the field of THz communications, THz imaging and atom trapping.

## 1. Introduction

Optical vortex beams (OVBs) carrying orbital angular momentum (OAM) hold promise for a higher data transmission capacity, have great applications in the field of light matter interactions [1,2], and have drawn vast attention in the past 30 years [3]. However, the research of terahertz (THz) wave-carrying OAM is less surveyed. Like the OVB, the THz vortex beam (TVB) has an azimuthal phase term *e^ilφ^*, where *l* is the topological charge (TC) and *φ* is the azimuthal angle [4,5]. TVBs have potential applications in high-speed THz communication, THz imaging, and atom trapping [6,7,8,9,10], and the generation of a TVB has been an important topic in the past few years [11]. Recently, by introducing electromagnetically induced transparency coupling to control nonlinear THz generation, a TVB with a different OAM was achieved [12]. Lu et al. reported that a hybrid nonlinear plasmonic metasurface incorporating indium tin oxide can be used to generate a TVB [13]. By designing all-silicon dielectric metasurfaces, Zhang et al. fabricated three TVB generators [14]. During these studies, to detect and characterize these generated TVBs, focusing and collimating are necessary [15,16]. However, traditional THz lenses are bulky and costly, and the generation and focus of a TVB, which are integrated into a single device, are an effect way to overcome this problem.

As an ultrathin, ultralight, and flat lens, Fresnel zone plates (FZPs) can directly focus a wave to the preferred position easily [17]. Recently, many THz generators integrated in FZPs have been proposed, and they can directly radiate focused THz waves or focused TVBs. However, most of these THz FZPs, which are based on nonlinear materials, e.g., plasmonic metasurfaces [18], InAs metasurfaces [19], and a patterned indium tin oxide film (ITO) [20], are amplitude FZPs, and only about 50% or less of the zones can generate a THz wave, resulting in a lower efficiency. Spintronic terahertz emitters (STEs), with the advantages of a low cost and a high performance, are considered as another potential terahertz (THz) source, and they have attracted immense attention in the past decade [21,22]. Recently, Chen et al. proved that an STE with an FZP could be produced, and a focused TVB was generated [23]. Unlike FZPs integrated in nonlinear materials, the π phase difference of the adjacent area of the FZP can be achieved by changing the direction of the deposited sequence of the STE. Therefore, it is a phase FZP, and the whole area of the STE can generate a focused TVB. However, they only obtained a TVB with a TC of *l* = 1. In addition, phase FZPs have a higher diffraction efficiency due to their larger numerical aperture (NA). Therefore, phase FZPs are a tight focusing element, and the field distributions of their focus are quite different from a common lens. To generate TVBs with different TCs and study the focused TVB more thoroughly, in this paper, we revisit STEs with a helical Fresnel zone plate (STE-HFZP).

A common STE consists of a ferromagnetic (FM) layer and a nonferromagnetic (NM) layer, and under the pump of a femtosecond laser pulse, the magnetized electrons in the FM layer will be excited to the state above the Fermi energy [21]. As a result of the FM layer and NM layer having different transport properties, an ultrafast spin current **j**_s_ will be induced, and then it will transform into an ultrafast charge current **j**_c_ due to the inverse spin Hall effect. The ultrafast charge current will radiate a THz wave with an electric field of **E**_THz_ ∝ *γ***j**_s_ × **M**/(|**M**|), where *γ* is the spin Hall angle and **M** is the magnetization of the FM layer, which can be changed by an external applied magnetic field [24]. Hence, by selecting NM layers with comparable magnitudes, but with opposite signs, two THz waves with a *π* phase difference can be generated.

## 2. Theoretical Design

For a usual FZP with focusing properties, the radius of the *n*-th ring is $r_{n}=\sqrt{n^{2}\lambda^{2}/4+n\lambda f}$, where *λ* is the working wavelength and *f* is the designed focal length [18]. To generate a focused TVB with a TC of *l*, the detour phase technique can be applied [25], where the location of each ring is slightly shifted and the radius of the *n*-th ring will be

(1) $$r_{n}=\sqrt{{\left(n\pi +l\varphi \right)}^{2}\lambda^{2}/4\pi^{2}+f\left(n\pi +l\varphi \right)\lambda /\pi }.$$

**Figure 1:**
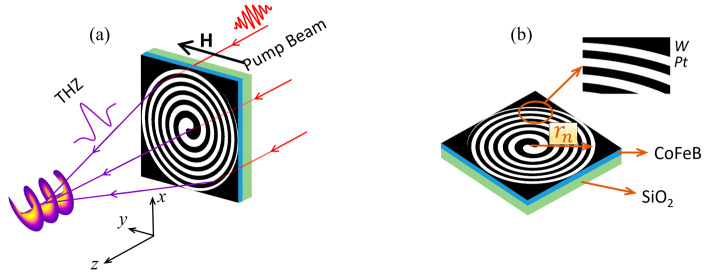
(**a**) Schematic of the STE-HFZP to generate a TVB with a TC of *l* = 1. *H*: the applied external magnetic fields along the *y* axis. The inset shows the wave front of the generated TVB. (**b**) Detail of the STE-HFZP. Here, the NM layers are selected as *W* and *Pt*, which are deposited on the FM layer (CoFeB) and arranged adjacently.

Figure 1a shows a schematic of an STE-HFZP based on the detour phase technique, and it has a helical cantilever. Under the pump of a femtosecond laser pulse, a focused TVB with a TC of *l* = 1 is generated, and the inset shows the wave front of the TVB. The THz signal has a peak when the thickness of the NM and FM layers is around 4 nm, respectively [15,21]; thus, the thicknesses of the NM and FM layers are selected as 4 nm, respectively. Figure 1b shows the detail of the STE-HFZP, where an FM layer (CoFeB) with a thickness of 4 nm is deposited on a SiO_2_ substrate [15]. Then, two helical NM layers (*W* and *Pt*) with the same thickness of 4 nm are deposited on the CoFeB film, and they are adjacently arranged. Here, we select *W* and *Pt* as the NM layer, because they almost have the same magnitude of *γ*, but their signs are opposite [26].

## 3. Results and Analysis

**Figure 2:**
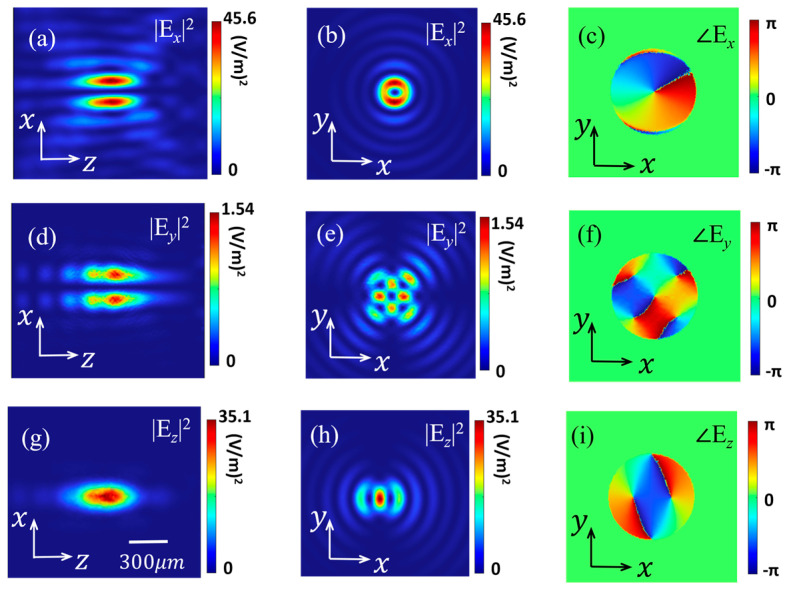
Calculated field intensities of the three electric components in the *y* = 0 mm plane (**a**,**d**,**g**) and the designed focal plane (*z* = 1 mm) (**b**,**e**,**h**). (**c**,**f**,**i**) show the phase profiles of the three electric components in the *z* = 1 mm plane.

In the next section, by using the finite element method, the performance of the designed STE-HFZP is surveyed. The generated transverse charge current **j**_c_ is proportional to the intensity of the local pump beam [21], and we assume that the generated **j**_c_ emits a THz wave with an electric field of 1 V/m, its polarization, which is perpendicular to the direction of the external magnetic field *H*, is along the *x*-axis. To reduce the time and computational memory costs while guaranteeing accurate simulations [27], an adequate three-dimensional geometry is modeled in COMSOL Multiphysics, and the scattering boundary conditions are adopted. The focal length of the STE-HFZP is *f* = 1 mm and its radius is *R* = 2.5 mm, corresponding to *NA* = [1 + (*f*/*R*)^2^]^−1/2^ = 0.93 [28]. The working frequency of the ST-FZPE is 1 THz, corresponding to *λ* = 300 μm. Figure 2 shows the calculated results, and Figure 2a,d,g shows the field intensities of the three electric components (*E_x_*, *E_y_* and *E_z_*) in the *y* = 0 mm plane. We find that the generated THz wave is focused and the largest electric field is *E_x_* at about 45.6 (V/m)^2^. Although the polarization of the generated THz beam is *x* polarization, the *y* and *z* components can also be found near the designed focus (*z* = 1 mm). However, most of the electric components in the focal plane is *E*_x_. In addition, the *z* component is larger than the *y* component, and it is comparable to the *x* component. These characteristics conform well to the tight focusing conditions of a high *NA* lens [29,30]. We can also estimate that the size of the focal spot is sub-wavelength (~300 μm). Figure 2b,e,h shows the intensities of the three electric components in the *z* = 1 mm plane, and we can find the *x* component has a donut shape, which is very similar to a vortex beam. Then, we calculate its phase profile (Figure 2c), and we can see that it has a helical wave front with a phase of *e^iφ^*. Hence, we can say that the STE-HFZP can directly emit a focused TVB with a TC of *l* = 1. We also calculate the intensities and phase profiles of the *y* and *z* components in the focal plane, as shown in Figure 2e,f,h,i, and these two components do not have a well-defined OAM due to their eccentric field distribution.

**Figure 3:**
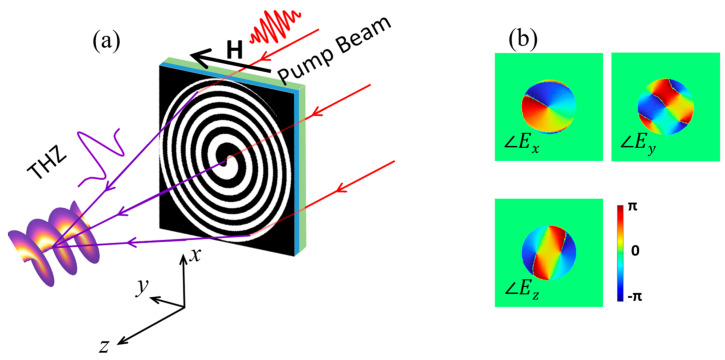
(**a**) Schematic of a STE-HFZP to generate a TVB with a TC of *l* = −1. (**b**) The phase profiles of the three electric components in the *z* = 1 mm plane.

**Figure A1:**
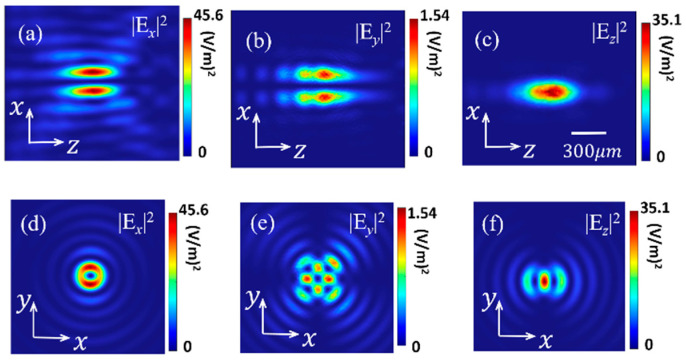
The field intensities of the three electric components with *l* = −1 in the *y* = 0 mm plane (**a**–**c**) and the designed focal plane (*z* = 1 mm) (**d**–**f**).

As we have shown before, the detour phase technique can be used to generate a TVB with a TC of *l* = 1. Thus, based on the detour phase technique, TVBs with other TCs can also be generated. Figure 3a shows a schematic of the STE-HFZP that generates a TVB with a TC of *l* = −1. Compared with Figure 1a, the helical direction of the STE is in the opposite direction, and the three electric components in the *y* = 0 mm plane and focal plane can be found in Figure A1 in the Appendix A. We can see that they have the same distributions as Figure 2. However, their phase profiles are opposite (Figure 3b). In Figure 3b, we can find the *x* component has a helical wave front with a phase of *e*^−*iφ*^, corresponding to a TC of *l* = −1. Like Figure 2f,i, the *y* and *z* components do not have well-defined OAMs as well, and their intensities are both lower than the *x* component. Therefore, *E_x_* with an OAM has a decisive effect on the light–matter interaction.

**Figure 4:**
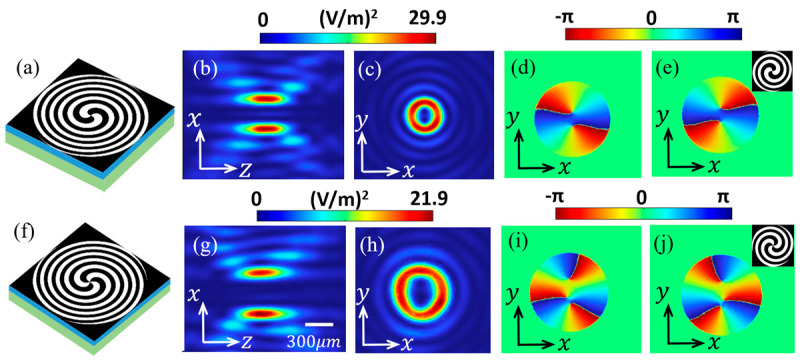
The STE-HFZP that generates TVBs with TCs of *l* = 2 (**a**) and 3 (**f**), respectively. The field distribution of the *x* component in the *y* = 0 mm plane (**b**,**g**) and *z* = 1 mm plane (**c**,**h**). The phase profile of the focused TVB (**d**,**e**,**i**,**j**). The insets in (**e**,**j**) show an STE-HFZP with *l* = −2 and −3, respectively.

**Figure A2:**
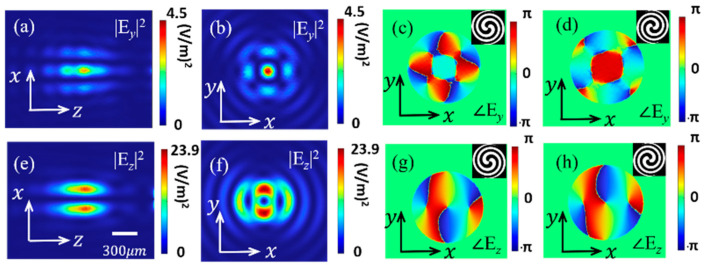
The intensities of electric fields *E_y_* and *E_z_* of the focused THz with *l* = 2 in the *y* = 0 mm plane (**a**,**e**) and the *z* = 1 mm plane (**b**,**f**). The phase profiles of *E_y_* and *E_z_*. The insets in figure (**c**,**d**,**g**,**h**) show the corresponding STE-HFZPs, and (**c**,**g**) correspond to *l* = 2 and (**d**,**h**) correspond to *l* = −2.

We also calculate the possibility of STE-HFZP generating TVBs with higher TCs. Figure 4 shows the STE-HFZP that generates TVBs with TCs of *l* = 2 and 3, and the corresponding STE-HFZP can be designed according to Equation (1). As shown in Figure 4a, the STE-HFZP has two helical cantilevers, and the generated THz wave is focused as shown in Figure 4b,c. Figure 4b,c shows the field intensities of *E_x_* in the *y* = 0 mm plane and *z* = 1 mm plane, respectively, and we can find they also have a donut shape. Compared with Figure 2, we can find the radius of the ring is increased. The focused *x* component also has a helical wave front with a phase of *e^i2φ^*, as shown in its phase profile in Figure 4d, and its TC is *l* = 2. The electric field and phase profile of the other two components of the focused THz wave can be found in Figure A2 in Appendix A. We can see that they are weaker than the *x* component, and they do not have well-defined OAMs as their phase profiles show. If we selected *l* = −2 in Equation (1), the direction of the two helical cantilevers will be reversed, as shown in the inset in Figure 4e, and a focused TVB with a TC of *l* = −2 is generated, as shown in the phase profile in Figure 4e. The field intensities of the three components are the same as the STE-HFZP with *l* = 2, and are not shown, while their phases are opposite, as shown in Figure A2 in Appendix A.

**Figure A3:**
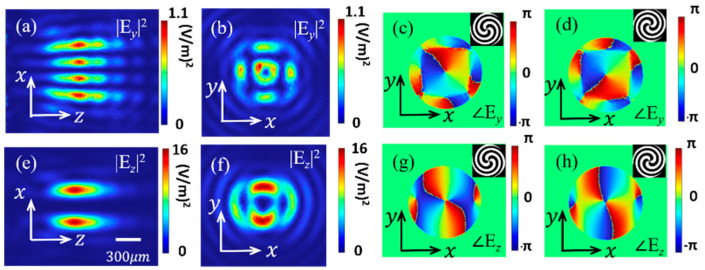
The intensities of electric fields *E_y_* and *E_z_* of the focused THz with *l* = 2 in the *y* = 0 mm plane (**a**,**e**) and the *z* = 1 mm plane (**b**,**f**). The phase profiles of *E_y_* and *E_z_*. The insets in figure (**c**,**d**,**g**,**h**) show the corresponding STE-HFZPs, and (**c**,**g**) correspond to *l* = 3 and (**d**,**h**) correspond to *l* = −3.

When *l* = 3 is selected in Equation (1), a focused TVB with a TC of *l* = 3 can be obtained. Figure 4f shows the STE-HFZP with *l* = 3, and it has three helical cantilevers. The generated THz beam is focused as shown in Figure 4g,h and Figure A3 in Appendix A. We find that the *x* component has a helical phase term of *e^i3φ^*, corresponding to a TC of *l* = 3 as shown in Figure 4i. Figure 4g,h shows the distribution of *E_x_* in the *y* = 0 mm plane and the *z* = 1 mm plane, respectively. Compared with Figure 4c, the radius of the ring is further enlarged. More importantly, *E_x_* is also larger than the other two components, which do not have a well-defined OAM. When the helical direction of the three helical cantilevers of the STE-HFZP is reversed, as shown in the inset in Figure 4j, a focused TVB with a TC of *l* = −3 is generated, as shown in the phase profile in Figure 4j. We should point out that the field intensities of the three components are the same as the STE-HFZP with *l* = 3, and they are neglected. Similarly, the phase profiles of the other two components are opposite to *l* = 3, as shown in Figure A3 in Appendix A. To generate TVBs with higher TCs and change the focus of the target frequency, we only need to change the pattern of the STE-HFZP according to Equation (1).

**Figure 5:**
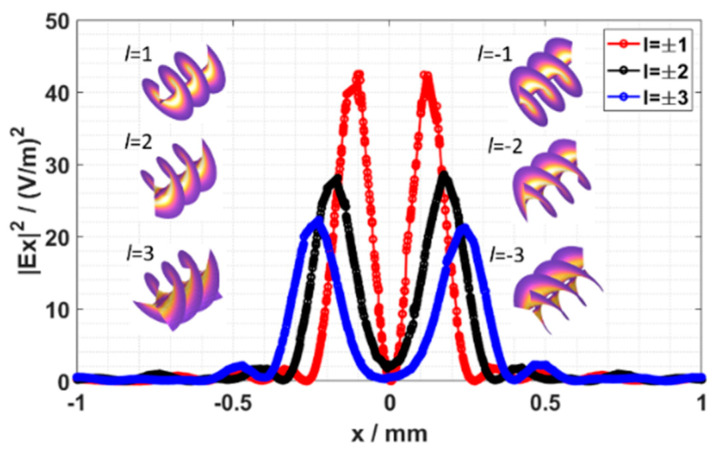
The line scans of the center of |*E_x_*|^2^ in the *z* = 1 mm plane with *l* = ±1, ±2 and ±3, respectively. The insets show the helical phase front of focused *E_x_*.

We have seen that with the increase in the TC, the radius of |*E_x_*| increases. To quantitatively analyze the radius of the ring, the line scans of the center of |*E_x_*| in the *z* = 1 mm plane are plotted and shown in Figure 5. The insets show the helical wave front of the focused *E_x_* with TCs of *l* = ±1, ±2 and ±3, respectively. These TVBs all have donut shapes, while their phases are opposite. It is clearly shown that the radii of the three rings are about 123 μm, 175 μm and 243 μm, respectively. This phenomenon is in good agreement with the property of a conventional vortex beam, where the ring size has a strong dependence on the TC [31].

## 4. Conclusions

In conclusion, in this paper, according to the detour phase technique, we design a series of STE-HFZPs to directly generate TVBs with different TCs. These STE-HFZPs are composed of two helical *W* and *Pt* layers, which are deposited on a CoFeB film. Due to Pt and W almost having the same magnitude of spin Hall angle *γ*, while their signs are opposite, a *π* phase difference in the adjacent area of the STE-HFZPs is achieved. As a result, the generated TVBs are self-focusing. The field distributions and phase profiles of generated TVBs with TCs of *l* = = ±1, ±2 and ±3 as three examples are calculated. We show that focused TVBs with higher TCs can also be generated from this method, and the components of the focused electric field with OAM make up the majority of the intensity. Compared with common THz lenses, these STE-HFZPs have a high NA, achieving subwavelength focal spots. These characteristics of the STE-HFZP may have potential applications in the field of THz communications, THz imaging and atom trapping.

## Data Availability

The data that support the findings of this study are available from the corresponding author upon reasonable request.
